# Exploring the acute cardiovascular effects of Floatation-REST

**DOI:** 10.3389/fnins.2022.995594

**Published:** 2022-12-09

**Authors:** M. C. Flux, Thomas H. Fine, Tate Poplin, Obada Al Zoubi, William A. Schoenhals, Jesse Schettler, Hazem H. Refai, Jessyca Naegele, Colleen Wohlrab, Hung-Wen Yeh, Christopher A. Lowry, Jason C. Levine, Ryan Smith, Sahib S. Khalsa, Justin S. Feinstein

**Affiliations:** ^1^Department of Psychology and Neuroscience, University of Colorado Boulder, Boulder, CO, United States; ^2^Department of Psychiatry, University of Toledo College of Medicine, Toledo, OH, United States; ^3^Laureate Institute for Brain Research, Tulsa, OK, United States; ^4^Department of Electrical and Computer Engineering, The University of Oklahoma, Tulsa, OK, United States; ^5^The University of Tulsa, Tulsa, OK, United States; ^6^Children’s Mercy Research Institute, Kansas City, MO, United States; ^7^University of Missouri-Kansas City, Kansas City, MO, United States; ^8^Float Research Collective, Kihei, HI, United States

**Keywords:** anxiety, interoception, autonomic, heart rate variability, blood pressure

## Abstract

The central nervous system (CNS) exerts a strong regulatory influence over the cardiovascular system in response to environmental demands. Floatation-REST (Reduced Environmental Stimulation Therapy) is an intervention that minimizes stimulation from the environment, yet little is known about the autonomic consequences of reducing external sensory input to the CNS. We recently found that Floatation-REST induces a strong anxiolytic effect in anxious patients while paradoxically enhancing their interoceptive awareness for cardiorespiratory sensations. To further investigate the physiologic nature of this anxiolytic effect, the present study measured acute cardiovascular changes during Floatation-REST using wireless and waterproof equipment that allowed for concurrent measurement of heart rate, heart rate variability (HRV), breathing rate, and blood pressure. Using a within-subjects crossover design, 37 clinically anxious participants with high levels of anxiety sensitivity and 20 non-anxious comparison participants were randomly assigned to undergo a 90-min session of either Floatation-REST or an exteroceptive comparison condition that entailed watching a relaxing nature film. Measures of state anxiety and serenity were collected before and after each session, while indices of autonomic activity were measured throughout each session. HRV was calculated using both time-series and frequency domain analyses. Linear mixed-effects modeling revealed a significant main effect of condition such that relative to the film condition, Floatation-REST elicited significant decreases (*p* < 0.001) in diastolic blood pressure, systolic blood pressure, breathing rate, and certain metrics of HRV including the standard deviation of the interbeat interval (SDNN), low-frequency HRV, and very low-frequency HRV. Heart rate showed a non-significant trend (*p* = 0.073) toward being lower in the float condition, especially toward the beginning of the session. The only metric that showed a significant increase during Floatation-REST was normalized high-frequency HRV (*p* < 0.001). The observed physiological changes were consistent across both anxious and non-anxious participants, and there were no significant group by condition interactions. Blood pressure was the only cardiac metric significantly associated with float-related reductions in state anxiety and increases in serenity. These findings suggest that Floatation-REST lowers sympathetic arousal and alters the balance of the autonomic nervous system toward a more parasympathetic state.

**Clinical trial registration:**

[https://clinicaltrials.gov/show/NCT03051074], identifier [NCT03051074].

## Introduction

Floatation-REST (Reduced Environmental Stimulation Therapy) reduces sensory input to the nervous system through the act of floating supine in a pool of water supersaturated with magnesium sulfate (Epsom salt). The float experience is calibrated so that visual and auditory stimulation is minimized, the temperature of the air and water is thermoneutral (i.e., matched to skin temperature), and the proprioceptive effects of gravity on muscle tension are significantly attenuated by the saltwater (which is calibrated to a specific gravity of 1.25 to allow for effortless floating, where approximately half of the body is floating above the surface of the water and the other half is submerged under the water). Floatation-REST was developed by Dr. John C. Lilly and Glenn Perry in the early 1970’s as a substitute for whole-body water immersion, a technique that was first used by Dr. Lilly and Dr. Jay Shurley in the 1950’s as a way to explore how the central nervous system might respond to an environment devoid of external sensory input (Shurley, 1960; Lilly, 1977). By the late 1970’s, several researchers began exploring Floatation-REST’s physiological and psychological effects (Fine and Turner, 1982; Suedfeld et al., 1983), and a meta-analysis of 27 studies published between 1983 and 2002 found large effect sizes across a population of mostly healthy subjects, including beneficial changes in physiological variables (e.g., lower blood pressure and cortisol) and increased levels of relaxation and subjective well-being (Van Dierendonck and Nijenhuis, 2005).

Specifically related to cardiovascular changes that occur as the result of Floatation-REST, several studies have reported significant reductions in both systolic and diastolic blood pressure when comparing measurements taken before and after a float session (Jacobs et al., 1984; Turner et al., 1989). More recently, our lab has found significant blood pressure reductions during the actual float session, with diastolic blood pressure showing the largest decrease when compared to watching a nature documentary (Feinstein et al., 2018b) or lying supine in a zero-gravity chair (Khalsa et al., 2020). Two previous studies explored the effects of Floatation-REST on heart rate (Forgays and Belinson, 1986; O’Leary and Heilbronner, 1990) with variable results. Forgays and Belinson (1986) recorded heart rate before, during, and after Floatation-REST over the course of three sessions and found that heart rate was highest before floating, steadily fell during the float, and then rebounded toward the end of the float session. The study, however, was limited by the lack of a control condition. O’Leary and Heilbronner (1990) included a supine control condition that entailed lying on a cot and measured changes in heart rate and blood pressure using a pre/post design, and found that Floatation-REST significantly decreased both metrics as compared to the control condition. Heart rate and blood pressure are key variables reflecting the operation of the autonomic nervous system (ANS). Breathing rate and heart rate variability (HRV) – i.e., the fluctuation in time between heartbeats – have also emerged as important variables to assess ANS function (Shaffer and Ginsberg, 2017; Nakao, 2019). Many mental health conditions appear to have an altered pattern of HRV, including major depression (Rottenberg, 2007; Kemp et al., 2010) and anxiety (Chalmers et al., 2014), with patients often showing significantly lower time domain measurements of HRV as well as decreased high-frequency HRV. Yet, to date there has been no research measuring breathing rate or HRV during Floatation-REST.

A core goal of the current study is to understand how the ANS responds when the CNS is exposed to an environment with minimal exteroceptive stimulation and metabolic demand. This window into ANS functioning in the absence of sensory stimulation could provide a novel paradigm for measuring an individual’s true physiologic baseline, one that is untethered from the influences of the external world. However, the baseline state engendered by floating is not affectively neutral, as prior research has shown that the float experience induces a state of serenity and decreased anxiety, effects that were most pronounced in clinically anxious patients (Feinstein et al., 2018a,b; Khalsa et al., 2020). Notably, all of the patients had high levels of anxiety sensitivity, which refers to one’s fear of experiencing anxiety-related symptoms and sensations, especially those arising from within the body (Taylor, 2014). Interestingly, the float environment was found to enhance awareness for interoceptive sensation, especially sensations emanating from the cardiorespiratory system (Feinstein et al., 2018b; Khalsa et al., 2020). This presents a paradox, as one might expect individuals with high anxiety sensitivity to find the conscious experience of cardiorespiratory sensations to be anxiety inducing (rather than reducing). This in turn raises the intriguing question as to how patients with high levels of anxiety sensitivity find serenity in an environment which amplifies awareness for the very sensations most commonly linked to the experience of anxiety.

A deeper understanding of the neurophysiological processes underpinning floating’s anxiolytic effect could have important clinical implications. In a prior study (Feinstein et al., 2018b), we theorized that the float environment may weaken the aversive association between visceral sensations and anxiety through a process of reciprocal inhibition (Wolpe, 1958), culminating in the formation of a new association, one that links the experience of cardiorespiratory sensations to a state of relaxation instead of anxiety. Unfortunately, very little is known about the physiological correlates of a float-induced state of relaxation. From a neural perspective, we have shown that Floatation-REST leads to decreased functional connectivity between the posterior insula, somatosensory cortices, and the default-mode network, with greater reductions in connectivity associated with higher levels of post-float serenity (Al Zoubi et al., 2021). However, from a cardiovascular perspective, there has not yet been any float studies linking the physiological response to subjective changes in anxiety and serenity.

The present study aims to add to this prior research by more thoroughly characterizing the cardiovascular changes induced by Floatation-REST and exploring whether any of the physiological changes are associated with its anxiolytic effect. Wireless and waterproof equipment was employed to measure heart rate, HRV, breathing rate, and blood pressure during a 90-min session of Floatation-REST, as well as during an exteroceptive comparison condition that entailed watching a relaxing nature film for an equivalent amount of time. Both conditions can be conceptualized as capturing an extended baseline state of cardiovascular functioning during which participants remained still, without movement or speech, over a 90-min period of time. Whereas the float condition measures the baseline physiology in an environment lacking exteroceptive stimulation, the film condition measures the baseline physiology in an environment with relatively neutral audiovisual stimulation. Thus, a within-subjects design was chosen to allow for the direct comparison of baseline cardiovascular functioning while one is in a stimulated versus unstimulated environment. All participants completed both conditions in a randomized order with approximately 1 week in between the float and film session. In addition, all participants floated one time prior to this experiment in order to acclimate them to the float environment. The participants were comprised of clinically anxious patients with high levels of anxiety sensitivity, most of whom were part of a prior study that only reported blood pressure changes (Feinstein et al., 2018b). We also recruited a smaller sample of demographically matched non-anxious comparison participants to explore whether they showed any notable differences in their physiological response. Since this was the first Floatation-REST study to concurrently measure heart rate, HRV, breathing rate, and blood pressure during the float experience, the analysis is largely exploratory in nature. Based on the relatively small body of prior research investigating physiological changes during Floatation-REST, we hypothesized that floating relative to the film condition would shift the balance in ANS functioning toward a state of physiological relaxation characterized by significant decreases in blood pressure, heart rate, and breathing rate, along with significant increases in HRV parameters related to the engagement of the parasympathetic nervous system including high-frequency HRV. We also predicted that the cardiovascular changes induced by Floatation-REST would be related to greater levels of serenity and lower levels of anxiety, helping to parse the physiological mechanisms by which floating exerts its anxiolytic effect.

## Materials and methods

All study procedures were approved by the Western Institutional Review Board, and all participants provided written informed consent prior to participation. The trial was registered on ClinicalTrials.gov, and this study was part of a larger project examining the subjective, physiological, and neural effects of Floatation-REST.

### Sample characteristics

There were 37 anxious participants who met inclusion and exclusion criteria and underwent the float and the film conditions (Supplementary Figure 3 and Supplementary Table 1). All anxious participants met criteria for one or more anxiety disorders, spanning the spectrum of different anxiety and stress-related conditions, with a mix of comorbidities, including generalized anxiety disorder (*n* = 20), social anxiety disorder (*n* = 13), panic disorder (*n* = 11), agoraphobia (*n* = 8), and posttraumatic stress disorder (*n* = 13). Most of the anxious participants also had comorbid major depressive disorder (*n* = 35). Table 1 provides additional details about subject demographics and baseline level of functioning. Two thirds of the participants (*n* = 23) were stably medicated (for 6 weeks or longer) on one or more psychotropic medications. Medication-related effects were mitigated by excluding subjects who recently began taking a new medication and by using a within-subjects design where every subject served as their own control. At the start of this study, the anxious participants were all acutely anxious and depressed, with average group scores well above the clinical range of severity (Overall Anxiety Severity and Impairment Scale (OASIS) score = 9.8; Patient Health Questionnaire nine-item depression scale score (PHQ-9) = 11.9). The anxious sample had high levels of anxiety sensitivity [average Anxiety Sensitivity Index-3 (ASI-3) total score = 29.0] as well as marked impairment in social and occupational functioning (average total disability score on the Sheehan Disability Scale = 14.1). We also recruited 20 non-anxious comparison participants (Supplementary Figure 4 and Supplementary Table 2) who were demographically matched to the anxious sample (Table 1). These participants had no history of any neurological or psychiatric illness and were not on any psychotropic medications. At the start of this study, the comparison participants were not acutely anxious or depressed (average OASIS score = 1.6; average PHQ-9 score = 2.5) and had low levels of anxiety sensitivity (average ASI-3 total score = 5.8).

**TABLE 1:** Participant demographics and baseline functioning.

	Anxious participants	Non-anxious participants
Sample size	37	20
Age	37.0 (11.4)	37.4 (11.3)
Sex, male/female	14/23	7/13
Medicated participants	23	0
Anxiety sensitivity (ASI-3)	29.0 (11.8)	5.8 (3.7)
Anxiety severity (OASIS)	9.8 (3.8)	1.6 (2.0)
Depression severity (PHQ-9)	11.9 (5.6)	2.5 (3.0)
Level of disability (SDS)	14.1 (7.9)	1.5 (3.0)

The total or average scores are presented for each metric. Numbers inside parentheses represent the standard deviation. ASI-3, Anxiety Sensitivity Index-3; OASIS, Overall Anxiety Severity and Impairment Scale; PHQ-9, Patient Health Questionnaire nine-item depression scale; SDS, Sheehan Disability Scale.

### Participant recruitment

Participants were recruited from a prior study (Feinstein et al., 2018a) where they underwent a single 60-min float session (without any physiological measurements) to help acclimate them to the float environment. On average, there was a 2-month gap between completing the previous study and the current study. In the previous study, participants were recruited through the Tulsa 1000 (T1000) database maintained at the Laureate Institute for Brain Research (LIBR). The T1000 is a naturalistic study that aims to recruit and longitudinally follow ∼1,000 treatment-seeking individuals from the local community, many of whom have anxiety and/or depression (Victor et al., 2018). Each participant received the Mini-International Neuropsychiatric Interview (MINI) version 6.0, and all psychiatric diagnoses were confirmed following review of the clinical history by a board-certified psychiatrist.

The specific inclusion and exclusion criteria (Supplementary Table 1) for recruiting anxious participants from the T1000 database into the initial float study targeted individuals with very high levels of anxiety sensitivity (defined as an ASI-3 total score ≥ 30) across the spectrum of different anxiety and stress-related disorders, many with comorbid unipolar depression. Participants were invited back to participate in the current study so long as they continued to present with at least a moderate degree of anxiety severity during the initial float study, defined as an OASIS score ≥ 6. All clinical self-report measures were re-administered at the start of this study, and the updated scores are presented in Table 1. Since the T1000 is a naturalistic study based on a community sample, we allowed participants who were stably medicated into the study. However, we added exclusion criteria for more severe forms of psychopathology and substance use in order to minimize potential safety risks.

### Study design

**FIGURE 1:**
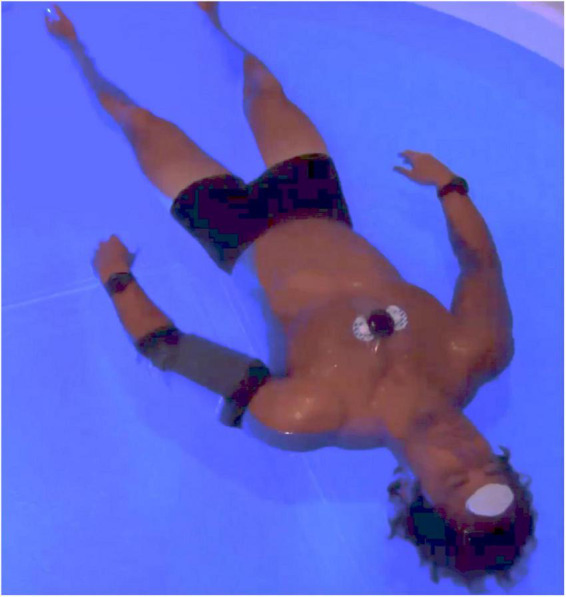
Concurrent physiological measurement during Floatation-REST (Reduced Environmental Stimulation Therapy). In order to collect physiological data during the float session, small non-invasive wireless sensors were attached to the participant. To measure heart rate, breathing rate, and HRV, a Zephyr BioPatch ECG system was attached to the chest with a layer of waterproof Tegaderm. To measure blood pressure, a QardioArm monitor was placed around the upper left arm with a waterproof cast. We also collected electroencephalography (EEG) using a wireless system that was placed on the forehead, in addition to accelerometry using accelerometers attached to each wrist; these data will be part of a separate publication. Of note, participants floated without any clothing on. The same sensors and setup were also used to collect physiological data during the film condition.

The protocol entailed a randomized controlled trial using a within-subjects crossover design. Participants were randomly assigned to complete a 90-min session of Floatation-REST (referred to as the float condition) or a 90-min session of an exteroceptive comparison condition (referred to as the film condition) that entailed watching a nature documentary from the BBC Planet Earth series (Attenborough and Fothergill, 2006). After completion of one condition, participants crossed over to the other condition approximately 1 week later (average time between conditions was 8 days), with both conditions scheduled to occur at the same time of day for each participant. The randomization sequence was predetermined using a 1:1 allocation ratio, and the study used an open-label design with no blinding or concealed allocation. Self-report measurements were recorded on a computerized tablet before and after each condition. Wireless and waterproofed physiological sensors were attached to the participant prior to the start of each float and film session (Figure 1).

### Floatation-REST condition

All float sessions occurred in an open circular fiberglass pool custom-designed for research purposes by Floataway (Norfolk, United Kingdom). The open circular float pool was 8 feet in diameter and contained 11 inches of water saturated with ∼1,800 pounds of USP grade Epsom salt (magnesium sulfate), creating a dense saltwater solution maintained at a specific gravity of ∼1.25, allowing participants to effortlessly float on their back. Since the pool had no enclosure, participants could freely enter and exit at any time. The room around the pool was constructed to be waterproof, soundproof, lightproof, and temperature-controlled (described in greater detail below). Silent heaters were placed under the pool to maintain the water at a constant temperature and a dedicated heating, ventilation, and air conditioning system maintained the air at a constant temperature. The temperature of the water and air approximated the surface temperature of the skin (∼95.0°F) and could be adjusted remotely by the experimenter in a nearby control room. An intercom system allowed the participant to freely communicate with the experimenter throughout the float session should any issues arise, and specialized speakers placed around the perimeter of the pool allowed the experimenter to communicate with the participant and play music to signal the end of the session. While floating, a blue LED light remains illuminated in the background and could be turned off by the participant using an air switch. Once triggered, the air switch activates an infrared wave detection system so that participants can turn the blue light on and off simply by waving their arm in the air while floating. The infrared wave detection system was linked to a digital clock, allowing for the automated calculation of the total amount of time that a participant was floating with the lights completely off.

The float pool and surrounding room were specially engineered to minimize all sensory signals from visual, auditory, olfactory, gustatory, thermal, tactile, vestibular, gravitational and proprioceptive channels. Visual stimulation was minimized by building an entry door and gasket system which expunged all sources of outside light. In addition, there were no windows inside the float room, and the adjacent room contained a private bathroom that also had no windows, and no lights (which were automatically shut off during the float itself). Thus, when the entry door to the float room was sealed and the blue LED light inside the pool was turned off, the float room was completely dark. Auditory stimulation was minimized by constructing the float room using multiple layers of sound dampening walls with thick insulation and added soundproofing material, restricting most outside airborne sound from entering the room. Structural sounds transmitted *via* vibrations in the floor were minimized by having the float pool rest on a bed of 48 butyl rubber springs, effectively isolating the pool from the building and preventing structure-borne noises from entering the water. Olfactory stimulation was minimized by using only unscented cleaning products and having the participant shower beforehand to help remove body odors. In addition, the water disinfection system used a combination of ultraviolet light and 35% hydrogen peroxide which does not emit any odors during the oxidative process. Gustatory stimulation was minimized by having participants eat several hours before the float, while refraining from eating and drinking during the float. Thermal stimulation was minimized by setting the temperature of the water and the air to closely match the temperature at the surface of the skin, which is typically a few degrees cooler than core body temperature. All temperature sensors were calibrated using a Thermoworks precision thermometer (Utah, USA) certified by the National Institute of Standards and Technology (NIST). Throughout each float session, the water temperature was maintained at 95.0°F (±0.3°F) and the air temperature at the rim of the pool was maintained at 93.5°F (±0.5°F), slightly lower than the water temperature based on the relative humidity in the air. This temperature setting helped minimize the need for thermoregulation while reducing the perceptual boundary between air, body, and water. Specific gravity of the water was calibrated using an H-B Instrument Polycarbonate Hydrometer (Pennsylvania, USA) that was NIST calibrated to achieve an accuracy within 0.002. The density of the water was maintained at a specific gravity between 1.25 and 1.26 for all float sessions. The body’s immersion in this dense saline solution minimized stimulation from tactile, vestibular, gravitational, and proprioceptive channels by buffering the body against the forces of gravity and allowing the individual to effortlessly float on their back in a state of stillness. The importance of “stillness” was also emphasized during the pre-float instruction set, further helping to minimize both movement and speech.

All participants were instructed that they could float “for up to 90 min” and could stop floating at any time. The following script was read prior to the float session: “Throughout the day, our brain and body are constantly bombarded by sensory information from the external world. In this study, we aim to understand what happens when you get a chance to disconnect from this constant stimulation by floating in an environment with reduced levels of light and sound, and reduced pressure on the spinal cord. While floating, try to find a place of stillness of both body and mind. You have complete control throughout the experience and can stop at any time. During the float we encourage you stay awake and when the float is over we will turn on some music for you. There is no rush, so please take your time exiting the pool.” No additional instructions were provided for how participants should spend their time during the float session. At the end of each float session (at ∼82 min), the song “Relax” by Blank & Jones (Relax Edition One, 2005) was played through the speakers, signaling to the participant that the float session was almost over.

### Film condition

The physiological changes during Floatation-REST were directly compared to an exteroceptive comparison condition that took place at the same time of day as the float session. The first 10 min entailed a baseline period during which participants remained with their eyes open while looking at a fixation cross on the screen. The remaining 80 min entailed watching three episodes (Great Plains, Jungles, and Seasonal Forests) from the BBC Planet Earth nature documentary series (2006) that were edited into a single film clip. The film contained pleasant and serene scenes of geographic landscapes and wildlife. Segments that had the potential to elicit intense emotional or physiological responses, such as depictions of violence or mating, were intentionally excluded. The film was presented on a computer monitor (21.5 × 15 inches) with speakers, and participants were seated upright in a stationary chair approximately 30 inches away from the monitor. The average room temperature during the film condition was 72.3°F (±1.6°F). As in the Floatation-REST condition, participants were encouraged to stay awake during the film and to try to find a place of stillness in order to minimize both movement and speech.

### Self-report measures

Participants completed all self-report measures on an Apple iPad using REDCap (Research Electronic Data Capture).^1^ Self-report measures were administered ∼30 min before and after completion of each condition, and across all measures participants were asked to rate how they felt “right now, in the present moment.” Pre- to post-float/film changes scores were computed for each participant.

### State-Trait Anxiety Inventory

The Spielberger State Anxiety Inventory (STAI) is a 20-item self-report questionnaire that assesses an individual’s anxiety at the present moment. Scores can range from 20 to 80 and items assess for the presence or absence of current anxiety symptoms. The STAI has excellent internal consistency and good convergent and discriminant validity (Spielberger et al., 1983).

### Serenity scale

Participants also completed the serenity scale from the Positive and Negative Affect Scale - Expanded Form (PANAS-X). The PANAS-X has high internal consistency, and good convergent, discriminant, and construct validity (Watson and Clark, 1999). Participants completed the PANAS-X serenity scale using the “at the present moment” instructions. The scale is comprised of three items: “How calm do you feel? How relaxed do you feel? How at ease do you feel?”

### Physiological data collection

Physiological measurements were recorded during each float and film session using a QardioArm blood pressure unit and a Zephyr BioPatch electrocardiogram (ECG) system (Figure 1). These devices were specifically chosen due to their small size, wireless sensors, and low voltage batteries which made the devices non-invasive and safe for use in the float pool’s aqueous environment. Blood pressure was measured using a QardioArm wireless monitor (Qardio Inc., San Francisco, California, USA) that is an FDA-approved automated sphygmomanometer which uses the Oscillometric method to achieve a measurement range of 40–250 mmHg and an accuracy of ±3 mmHg. The QardioArm has been clinically validated according to ANSI/AAMI/ISO 81060-2:2009 as well as the European Society for Hypertension International Protocol (O’Brien et al., 2010). The blood pressure cuff was positioned approximately 1 inch above the elbow, around the left upper arm, so that it was situated at the same level as the heart. During both conditions, participants were instructed to keep their arms positioned downward, resting along the side of their body. A LimbO Waterproof Protector (Limbo USA, Portland, Maine, USA) was placed over the QardioArm in order to prevent water from reaching the QardioArm during the float session. To ensure comparability across conditions, participants also wore the LimbO during the control condition. All blood pressure data were wirelessly transmitted in real-time *via* Bluetooth 4.0 to an iPad tablet located in the adjacent control room. Each blood pressure measurement took 30–60 s to complete and was initiated remotely by the experimenter using an application on the iPad. Blood pressure measurements were collected once every 10 min during the float and film conditions, with the first 3 measurements taken every 5 min in order to provide a higher temporal resolution for any potential changes occurring toward the beginning of a session. A small number of measurements (<3% of the total blood pressure measurements) were missing (due to a temporary loss in Bluetooth connectivity with the QardioArm) or were excluded if the reading was deemed an outlier based on previous guidelines that recommended excluding all measurements greater than 2 standard deviations from an individual’s average blood pressure during the float or film session (Lee et al., 1995). In addition, blood pressure data from one anxious participant was excluded due to a complete loss in Bluetooth connectivity during the float session. This left complete datasets from 56 participants (36 anxious and 20 non-anxious participants) where every participant had blood pressure data successfully collected in both conditions.

The Zephyr BioPatch was used to record ECG, heart rate, and breathing rate. The BioPatch is a CE-certified, FDA-approved Class II device that is water resistant up to 1 meter (IP67) and powered by a 3.7 V rechargeable lithium-ion battery (making it completely safe in the event that water did get in). Through its FDA approval, the BioPatch was shown to be equivalent to the Zephyr BioHarness 3.0, a predicate device. The reliability and validity of the BioHarness has been established in prior studies comparing the device to previously validated ECG devices such as the 12-lead Schiller AG AT-110 (Rawstorn et al., 2015). Zephyr Technologies has shown that the BioHarness 3.0 measures heart rate with a mean bias of 1.3 beats and SE of 0.29 beats as compared to Cortex BioPhysik MetaMax CPX 3 lead ECG system (Zephyr Technology, 2008). This was within the study’s validation criteria of mean bias and a standard error within 3 beats of the validated ECG system. In a review of ten validation studies of the BioHarness, the device was reported to have good construct validity with coefficients ranging from 0.74 to 0.99 when compared to gold standard devices (Nazari et al., 2018). The BioPatch device used in this study contains the same recording device, Zephyr BioModule, but is not secured by a chest strap. While the Bioharness utilizes the chest strap to monitor breathing rate, the BioPatch uses a proprietary impedance-based metric (Zephyr Technology, 2012; Russell et al., 2014). The BioPatch also contains a 3-axis accelerometer which was used to establish the start time of each float based on the moment of transition into a supine position.

The BioPatch uses a 2-lead ECG that is placed on the chest above the sternum using adhesive electrodes. To protect the device from water, it was covered with Tegaderm made by 3M, a transparent watertight medical dressing. Tegaderm also covered the BioPatch during the film condition to ensure comparability. Black electrical tape was used to cover a flashing LED light on the BioPatch, removing this extra source of visual stimulation. All data collected by the BioPatch in the film and float conditions were stored locally on the device and then transmitted to a computer after completion of the experiment. The BioPatch recorded ECG at 1,000 Hz and calculated the average heart rate and breathing rate at 1 Hz. The BioPatch also outputs heart rate confidence values that account for the signal-to-noise ratio of the ECG signal, with confidence values above 20% indicating a reliable signal. All time points below 20% confidence were excluded from the analysis (Zephyr Technology, 2016). If a float or film session contained over 30 min of ECG data under 20% confidence, then the entire dataset was excluded; this criterion ultimately led to the exclusion of 6 participants. In addition, a small amount of individual datapoints (<1% of the total BioPatch measurements) were excluded during manual inspection of the ECG data as described in the subsequent section.

### Physiological metric calculation

All analyses utilized physiological data from the first 80 min of the float and film session since music was played during the final 10 min of the float session and some subjects exited the pool before 90 min had elapsed. The average heart rate and breathing rate were calculated over 5-min bins based on the values outputted from the BioPatch each second. HRV metrics were also binned into 5-min intervals and extracted from time and frequency domains. Time domain metrics refer to measurements that quantify the amount of variability in the inter-beat interval, while frequency domain metrics explore the power of discrete frequency components, as measured *via* Fast Fourier Transformation or autoregressive modeling of the ECG signal. Initial standards for these measurements were developed by a task force in Malik et al. (1996). Among the time domain metrics are the standard deviation of the interbeat interval of normal sinus beats (SDNN), and the root mean square of successive differences between normal beats (RMSSD). The SDNN represents all of the cyclic components that go into variability during a recording period, while the RMSSD reflects changes in HRV that are vagally mediated and largely independent of respiratory influences (Laborde et al., 2017; Shaffer and Ginsberg, 2017). For time-domain analyses, we extracted the SDNN and RMSSD. For frequency-domain analyses, the HRV metrics were estimated based on the total and ratio power spectral densities within predefined frequency bands. Welch’s procedure was used to estimate the power spectral density from three frequency bands of interest as follows: the very low frequency (VLF) band [0.003–0.04 Hz], low frequency (LF) band [0.04–0.15 Hz], and high frequency (HF) band [0.15–0.4 Hz] (Ramshur, 2010). The measures of spectral power are reported in terms of the absolute VLF (aVLF), normalized LF (nLF), and normalized HF (nHF).

Electrocardiogram signals were first preprocessed using traditional signal processing including smoothing, detrending and scaling to achieve consistent and reliable peak detection. In addition, ECG points corresponding to low heart rate confidence intervals (<20% as the recommended threshold for usable data based on the BioPatch manual) were excluded from analysis prior to the peak detection phase. Due to the length of sessions (80 min), conducting quality assurance checks on the HRV data can be challenging. Thus, we designed and implemented a customized semi-automatic QRS complex detection algorithm in MATLAB to automatically annotate detected peaks on long ECG recordings. When the algorithm does not detect a peak within two seconds from the previous peak, the time period is annotated for manual inspection to determine where a peak occurs within the time frame. Manual inspection of each individual’s annotated ECG signal was conducted visually by research assistants to ensure the quality of detected peaks and ensure the exclusion of abnormal signals. The most prominent peaks outputted by the BioPatch were either the R or S wave from the QRS complex. Each dataset only had one prominent peak (either the R or S wave), thus, for each dataset, either the R or S peak was selected to compute the interbeat intervals (IBI) between detected peaks.

We utilized HRVAS (Ramshur, 2010), a Matlab-based HRV software package that has been utilized in dozens of other peer-reviewed papers to extract the aforementioned HRV metrics. The IBI series was divided into non-overlapping 5-min windows for each individual and processed by HRVAS to provide time-domain and frequency-domain analysis. Before calculating the time-domain metrics, HRVAS performs an ectopic interval detection and correction process to reduce analysis errors due to abnormal heartbeats. The frequency domain analysis also includes the same ectopic interval detection and correction process, but also undergoes a detrending and an IBI resampling process for the Welch periodogram analysis used here. These additional processes were necessary to ensure the stationarity of each IBI series as required by the power spectrum estimations and helped remove low-frequency trends and ensure that the series were regularly sampled.

### Statistical methods

All measures were analyzed by linear mixed-effects models (LMMs). HRV measures (including nHF, nLF, aVLF, SDNN, and RMSSD) were log transformed for use in parametric testing and LMMs as none met the criteria for normality. First, to assess the effects of floating on each physiological measure, we considered 3 main-effects: condition (float vs. film), group (anxious vs. non-anxious), and time (either presented as a continuous variable, or categorically). The analysis fitted separate LMMs with different fixed-effects: main-effects only and main-effects with all possible combinations of interaction terms. All LMMs included random intercepts to account for participant-specific variability. Bayesian information criterion (BIC) was used to identify the optimal models. Secondly, we evaluated the correlations between physiology and changes in state anxiety and serenity using LMM; each physiological measure was regressed on change scores of state anxiety and serenity, condition, and their 2-way interaction, along with time and group. All analysis was performed using RStudio version 1.0.136, using the R packages *lme4* (version 1.1-19) for LMM and the R package *lmerTest* (version 3.0-1) for calculation of degrees of freedom and *p*-values based on the Kenward–Roger method.

## Result

The anxious and non-anxious groups were closely matched based on age and sex (Table 1). All participants in both groups completed the float and film session. Most participants remained floating for the entire 90-min duration, with the exception of seven anxious and four non-anxious participants who exited the pool shortly after the music signaling the end of the float session started playing (∼85 min into the float). Thirteen anxious and seven non-anxious participants floated with the lights off throughout the entire session and the remaining participants all floated with the lights off for the vast majority of the session (80.5 min on average).

**FIGURE 2:**
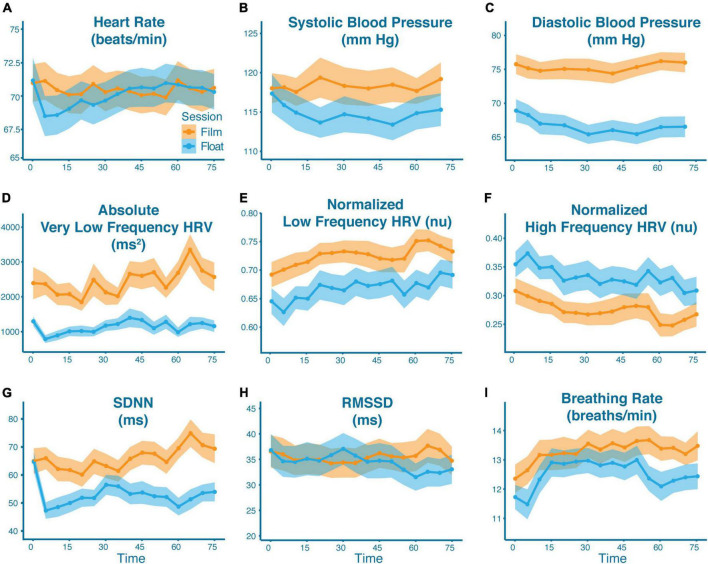
Cardiovascular effects of Floatation-REST as compared to the film condition. Mean physiological response as broken down by condition (blue = float; orange = film) across all participants for panel **(A)** heart rate, **(B)** systolic blood pressure, **(C)** diastolic blood pressure, **(D)** absolute very low frequency HRV, **(E)** normalized low frequency HRV, **(F)** normalized high frequency HRV, **(G)** SDNN, **(H)** RMSSD, and **(I)** breathing rate. The shaded region represents the standard error of the mean. The *x*-axis represents time (in minutes) since the start of the float or film. With the exception of blood pressure (which were single point measurements), data are graphed in 5-min bins such that timepoint 0 is the average from 0 to 5 min, and timepoint 75 is the average from 75 to 80 min. Significant differences were found between conditions for all variables except heart rate.

BIC selected models only containing the main effects of group (anxious vs. non-anxious), condition (float vs film), and linear time in predicting each individual cardiac metric. For every metric, with the exception of breathing rate, the term for group was not significant. As no significant effect of group was found for any of the cardiac metrics, Figure 2 displays the data for each condition collapsed across groups, with Table 2 reporting the overall means and effect sizes. The data broken down by each group is displayed in Supplementary Figure 1. Supplementary Figures 3, 4 show how many datasets were excluded for each physiologic metric. The study was powered to detect an f^2^ effect size of 0.133 at a power of 0.8 (Selya et al., 2012). This is between a small and medium effect size (small: f^2^ ≥ 0.02, medium: f^2^ ≥ 0.15, and large: f^2^ ≥ 0.35).

**TABLE 2:** Mean (SD) and between-condition effect sizes for each physiologic metric.

	Float	Film	Cohen’s *d*
Heart rate	70.01 (9.88)	70.41 (9.62)	0.04
Breathing rate	12.52 (3.14)	13.28 (3.27)	0.27
SDNN	52.85 (23.02)	65.92 (29.17)	0.59
RMSSD	34.42 (20.51)	35.49 (19.96)	0.05
nHF	0.33 (0.16)	0.28 (0.14)	0.49
nLF	0.67 (0.16)	0.73 (0.14)	0.49
aHF	608.46 (774.44)	548.06 (632.41)	0.09
aLF	1078.04 (1168.07)	1339.68 (1228.68)	0.28
aVLF	1126.95 (1168.07)	2431.97 (2452.27)	0.97
Systolic BP	114.91 (15.73)	118.31 (15.26)	0.22
Diastolic BP	66.76 (11.32)	75.30 (10.70)	0.84

For heart rate, there was a non-significant trending effect of condition (mean difference (diff): 0.4 beats per minute; *F*_(1,1486)_ = 3.20, *p* = 0.073) with lower heart rates in the float condition, and a non-significant trending effect of time (*F*_(1,1486)_ = 3.42, *p* = 0.065) with a gradual increase in heart rate over the course of both conditions. There were significant differences between conditions for breathing rate (diff: 0.76 breaths per minute; *F*_(1,1485)_ = 42.5, *p* < 0.001) with lower breathing rates in the float condition. There were also significant breathing rate differences between groups (diff: 1.41 breaths per minute; *F*_(1,46.0)_ = 4.49, *p* = 0.039), with higher breathing rates found among anxious participants in both conditions, as well as significant breathing rate differences over time (*F*_(1,1485)_ = 6.86, *p* = 0.009), with breathing rates increasing over the course of both conditions. For blood pressure, there were significant effects of condition for both systolic blood pressure (diff: 3.40 mm Hg; *F*_(1,944)_ = 42.5, *p* < 0.001), and diastolic blood pressure (diff: 8.54 mm HG; *F*_(1,945)_ = 598.3, *p* < 0.001), indicating lower blood pressure in the float condition as compared to the film condition.

In terms of HRV, all metrics showed significant effects of condition when comparing the float to the film. This included increased nHF (diff: 0.06; *F*_(1,1416.1)_ = 100.2, *p* < 0.001), decreased nLF (diff: 0.06; *F*_(1,1416.1)_ = 102.8, *p* < 0.001), decreased aVLF (diff: 1305.0; *F*_(1,1413.2)_ = 349.7, *p* < 0.001), decreased SDNN (diff: 13.1; *F*_(1,1414.1)_ = 198.5, *p* < 0.001), and decreased RMSSD (diff: 1.07; *F*_(1,1416.0)_ = 4.08, *p* = 0.043). Each metric also showed significant effects of time in both conditions (measured continuously), including a decrease in nHF over time (*F*_(1,1416.0)_ = 24.6, *p* < 0.001), an increase in nLF over time (*F*_(1,1416.0)_ = 24.2, p = 0.001), an increase in aVLF over time (*F*_(1,1413.1)_ = 14.4, *p* = 0.001), an increase in SDNN over time (*F*_(1,1414.0)_ = 5.20, *p* = 0.023), and a slight decrease in RMSSD over time (*F*_(1,1416.0)_ = 4.00, *p* = 0.046).

**FIGURE 3:**
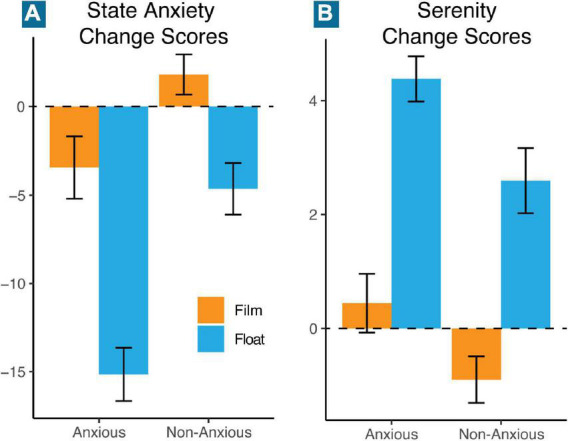
Mean change in state anxiety and serenity. Mean change scores from pre- to post-float/film were computed for each group and condition (orange = film; blue = float) for panel **(A)** state anxiety, and **(B)** serenity. Error bars represent the standard error of the mean.

**FIGURE 4:**
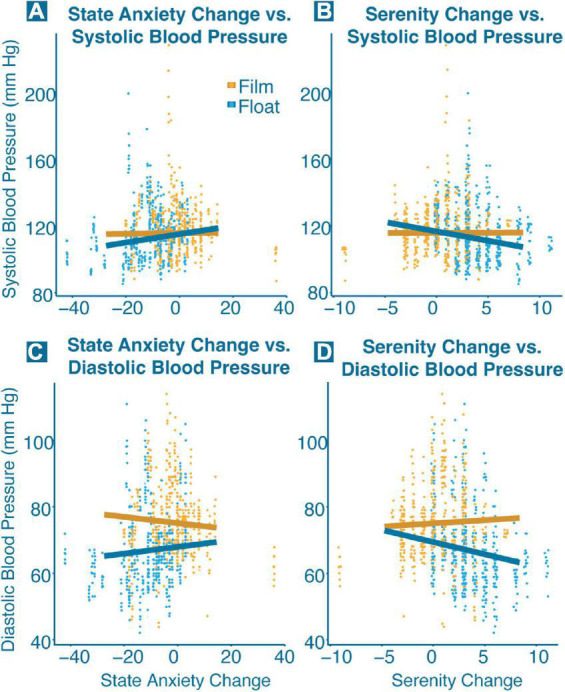
Significant interactions between blood pressure and change in state anxiety and serenity. Each point represents a single blood pressure measurement for one participant, and each trend line shows the correlation between blood pressure and change in state anxiety and serenity broken down for each condition (orange = film; blue = float). Significant interactions (*p* < 0.001) were found between **(A)** state anxiety change and systolic blood pressure, **(B)** serenity change and systolic blood pressure, **(C)** state anxiety change and diastolic blood pressure, and **(D)** serenity change and diastolic blood pressure.

In the second phase of analysis, we examined the relationship between physiology and changes in state anxiety and serenity. As compared to the film condition, Floatation-REST induced higher levels of serenity (*F*_(1,52.1)_ = 22.8, *p* < 0.001) and lower levels of state anxiety (*F*_(1,110.0)_ = 7.50, *p* = 0.007) across both groups (Figure 3); neither model contained significant group by condition effects. There were significant interactions between changes in state anxiety and condition for systolic blood pressure (*F*_(1,947.3)_ = 11.0, *p* = 0.001), diastolic blood pressure (*F*_(1,947.9)_ = 15.4, *p* < 0.001), and heart rate (*F*_(1,1482.9)_ = 21.4, *p* < 0.001). Simple slope analysis for the interaction predicting systolic blood pressure revealed a significant positive slope for the float condition (slope = 0.25, *p* < 0.001), and a non-significant slope for the film condition (slope = 0.02, *p* = 0.711), indicating that in the float condition, but not film condition, individuals who decreased in state anxiety tended to have lower systolic blood pressure (Figure 4). Simple slope analysis for the interaction predicting diastolic blood pressure revealed a significant positive slope for the float condition (slope = 0.10, *p* = 0.006) and a significant negative slope for the film condition (slope = −0.09, *p* = 0.010). This indicates that in the float condition, individuals who decreased in state anxiety tended to have lower diastolic blood pressure, but that this relationship was slightly reversed in the film condition (Figure 4). Simple slope analysis for the interaction predicting heart rate identified a significant positive slope for the film condition (slope = 0.16, *p* < 0.001), but a non-significant slope for the float condition (slope = 0.01, *p* = 0.788). This indicates that in the film condition, but not float condition, individuals who decreased in state anxiety tended to have lower heart rates during the condition (Supplementary Figure 2).

Interactions between changes in serenity and condition were found for breathing rate (*F*_(1,1512.4)_ = 13.5, *p* < 0.001), systolic blood pressure (*F*_(1,949.4)_ = 20.7, *p* < 0.001), and diastolic blood pressure (*F*_(1,950.0)_ = 27.7, *p* < 0.001). Simple slope analysis for the interaction predicting breathing rate identified a positive slope for the film condition (slope = 0.10, *p* = 0.019), and a more strongly positive slope for the float condition (slope = 0.305, *p* < 0.001). This indicates a counterintuitive relationship, such that greater increases in serenity were associated with higher breathing rates in both conditions (Supplementary Figure 2). Simple slope analysis for the interaction predicting systolic blood pressure revealed a significant negative slope for the float condition (slope = −1.12, *p* < 0.001) and a non-significant slope for the film condition (slope = 0.02, *p* = 0.906). Simple slope analysis for the interaction predicting diastolic blood pressure revealed a similar pattern, with a significant negative slope for the float condition (slope = −0.73, *p* < 0.001), and a non-significant slope for the film condition (slope = 0.19, *p* = 0.139). Both models for measures of blood pressure indicate that individuals who increased the most in serenity over the course of the float tended to have lower systolic and diastolic blood pressure during their float session (Figure 4).

## Discussion

This was the first study to investigate the cardiovascular and respiratory-related effects during Floatation-REST, an intervention which minimizes external sensory stimulation of the human nervous system. Using a randomized within-subjects crossover design, a combined cohort of 57 participants underwent a 90-min float session and watched a 90-min nature film. Relative to the film condition, Floatation-REST significantly lowered both diastolic and systolic blood pressure, as well as breathing rate. Heart rate showed a non-significant trend of being lower during the float condition, especially during the early phase of the float session. In the time domain of HRV, Floatation-REST resulted in a moderate decrease in SDNN and a smaller decrease in RMSSD. In the frequency domain of HRV, Floatation-REST increased nHF and decreased nLF and aVLF. Most of the physiological changes occurred within the first 15 min of the float, and often persisted for the remainder of the session (Figure 2). The effect sizes for the physiological changes were in the small to medium range, except for the reductions in diastolic blood pressure and aVLF, which were both large effects (Table 2). Notably, we did not observe any significant physiological differences between anxious and non-anxious comparison participants, save for the fact that the anxious participants had a higher breathing rate across both conditions. Overall, these results are generally consistent with Floatation-REST inducing lower sympathetic output and higher parasympathetic modulation but yield to a more dynamic and nuanced interpretation when viewed in the wider context of the intricate circuitry that serves to balance the ANS, including the influence of the baroreflex and respiratory sinus arrhythmia (RSA), which we discuss in more detail below.

The baroreflex is a feedback system that maintains blood pressure homeostasis. Mechanoreceptors present in the aortic and carotid sinuses respond to blood pressure, increasing firing rates in response to increased pressure and hypertension (Taylor et al., 2014; Ghitani and Chesler, 2019; Min et al., 2019). This signal, carried through either cranial nerve IX (glossopharyngeal nerve) or X (vagus nerve), synapses onto the nucleus of the solitary tract (NST) in the brainstem (Pilowsky and Goodchild, 2002). Activation of the NST both decreases sympathetic activity through brainstem projections to the intermediolateral nucleus of the spinal cord and increases vagal outflow. This in turn leads to a withdrawal of sympathetic activation from the heart’s pacemaker, from myocardial cells, and from vascular smooth muscle tissue. This results in a decrease in blood pressure due to decreased heart rate and vasodilation, allowing for a return to homeostasis. The opposite is also true, but in reverse. Hypotension leads to decreased activation of baroreceptors, leading to elevated sympathetic activity resulting in increased heart rate, vasoconstriction, and an increase in blood pressure.

When viewing ANS activity in the frequency domain, it was initially identified that the sympathetic output of the baroreflex corresponds to the “10-s rhythm” in humans (DeBoer et al., 1987), or the ∼0.1 Hz “Mayer wave” (Julien, 2006). Thus, the power of the 0.1 Hz band, which lies squarely in the LF band range, is presumed to primarily reflect the sympathetic activation component of the baroreflex, particularly at rest (McCraty and Shaffer, 2015). The current study’s finding of a significant decrease in nLF during Floatation-REST provides evidence supporting the notion that there was a decrease in sympathetic output from the baroreflex, which aligns well with the significant decrease in blood pressure observed during the float condition.

Somewhat complementary to the baroreflex is the RSA and the impact of breathing on HRV. As the name implies, this process is primarily related to respiration, leading to an elevation of heart rate during inspiration, and a reduction of heart rate during expiration (Berntson et al., 1993; Yasuma and Hayano, 2004). This process is driven by slowly adapting pulmonary stretch receptors localized to the tracheobronchial tree in the lungs (Schelegle, 2003). Excitation of slowly adapting pulmonary stretch receptors during inspiration sends signals through the vagus nerve to the NST, which then inhibits activity in the nucleus ambiguous, decreasing vagal output and resulting in a speeding up of the heart rate. The opposite is true in the case of expiration, with vagal output increasing, resulting in a slowing down of the heart rate. Because of this, the RSA is primarily a reflection of vagal variability, or vagal tone. Because vagal innervation of the heart’s pacemaker only involves two synapses after exiting the brainstem, as compared to three synapses for nerves of the sympathetic nervous system, this quicker activity is reflected primarily in the HF band of HRV, which is also referred to as the respiratory band for this reason (Shaffer and Ginsberg, 2017). While HF reflects the strength of this vagal component, its variation is reflected in RMSSD of the time domain. The current study’s finding of elevated nHF during Floatation-REST suggests increased vagal modulation, potentially due to the slower breathing observed during the float condition. This is consistent with the biological behavioral model that highlights the influence of breathing on vagal tone (Grossman and Taylor, 2007). Even though Floatation-REST involves placing an individual in an environment with reduced metabolic demand, mean breathing rates while floating remained within a normal range of approximately 12 breaths/min and only lowered an average of less than 1 breath/min as compared to the film condition. A complete understanding of the impact of breathing on vagal tone was limited by the omission of measuring tidal volume. Future studies may benefit from including this metric, as breathing rate and tidal volume produce independent and interactive effects (Hirsch and Bishop, 1981), and anxiety has been associated with increased respiration rate and decreased tidal volume in the lab (Gorman et al., 1988; Schwartz et al., 1996).

The responsiveness of HRV in the frequency domain as well as blood pressure can clearly be seen in the context of relaxation. For example, a variety of relaxation techniques have been connected to increases in HF-HRV and decreases in blood pressure (Benson et al., 1975; Terathongkum and Pickler, 2004). Similar to Floatation-REST, inwardly focused meditation has resulted in a decrease in LF-HRV and an increase in HF-HRV (Wu and Lo, 2008). A comparable pattern of HRV results was also found in a meta-analysis looking at the physiological effects of Tai Chi and Yoga (Zou et al., 2018). This aligns with the neurovisceral integration model, which was developed to help explain the interaction between peripheral cardiovascular physiology and cognitive function in relation to health outcomes (Smith et al., 2017). Because neural regulation of the heart involves so many different levels of integration, the heart’s behavior acts as a metric for health across levels, in everything from autonomic functioning to mental health.

With this added context, it may be possible to consider the frequency domain results as representative of a relaxation response. Elevation of nHF likely reflects increased vagal modulation as part of the RSA, which may be related to the non-significant decrease in heart rate found during the early part of the float session as well as the significantly decreased breathing rate found throughout the float session (though as a counterpoint, the decreased breathing rate observed during Floatation-REST is not nearly as low as the slow-paced breathing typically used to measure this effect in other experiments, e.g., Kapidžić et al., 2014 and Kromenacker et al., 2018). Decreases in nLF likely reflect reduced Mayer wave activity, and thus reduced baroreflex activity resulting in lower blood pressure. This may, in part, reflect prefrontal inhibition of sympathetic output normally coming from baroreflex activity (Julien, 2006; McCraty and Shaffer, 2015). While the significantly lower aVLF observed during Floatation-REST showed a large effect size, there is currently no consensus on a straightforward interpretation of this component. Some have suggested that it is related to parasympathetic activity and thermoregulatory vasomotor control (Fleisher et al., 1996) which would align with our findings, but more investigation is needed (Kleiger et al., 2005). Of note, the present frequency domain results, and the initial study hypotheses, would suggest a larger decrease in heart rate than what was found. However, this result could also reflect other compensatory mechanisms at play in maintaining heart rate in the face of the decreased blood pressure found during Floatation-REST. Interestingly, visual inspection of the heart rate plot (Figure 2) showed a dip in heart rate during the first 15 min that then returned to normal levels, indicating that such compensatory mechanisms could be relatively fast acting.

The HRV results in the time domain were somewhat unexpected, with both SDNN and RMSSD found to be significantly lower during Floatation-REST. SDNN reflects all the cyclic components responsible for variability in the period of recording, and RMSSD is a reflection of vagal tone, or the responsiveness of the vagus nerve signal to changing energetic needs from the environment that is mostly free of respiratory effects (Laborde et al., 2017). Other relaxation-based interventions have found increases in SDNN and RMSSD (e.g., Vinay et al., 2016). However, there are several differences between typical measures of time-based HRV and the current study. Firstly, this experiment measured cardiorespiratory activity continuously during the float session, rather than in a pre/post design. Leveraging the use of wireless and waterproof technology provided real-time estimates of psychophysiological activity and captured any regulatory adjustment to the float environment. Secondly, Floatation-REST reduces all external stimulation to an absolute minimum. Because of this, there is very little exteroceptive sensory information to trigger a varied autonomic response. Thus, it is possible that these experimental results speak to higher vagal modulation, as measured by elevated nHF and lower nLF, but a lack of variation within that response, as indicated by reduced SDNN and RMSSD, due to the fact that there were no external stimuli to respond to during Floatation-REST.

While the current study did not find any interactions between HRV metrics in either the time or frequency domain and changes in anxiety, there were significant interactions between blood pressure and changes in both state anxiety and serenity. In each case, individuals with lower blood pressure during floatation showed the greatest decreases in state anxiety and the greatest increases in serenity, while this association was not found in the film condition. This suggests that the blood pressure reductions found during Floatation-REST may be playing an important role in the anxiolytic effect. Other interaction results were more perplexing. For example, there was a positive relationship between anxiety change and heart rate for the film condition, but no significant relationship for the float condition. During the film, individuals who showed the largest decrease in state anxiety tended to have lower heart rates while watching the film. The lack of a significant relationship during the float condition is unexpected but may align with the abovementioned hypothesis that another compensatory mechanism is maintaining a normal heart rate during floatation. Alternatively, it is possible that the magnitude of the small dip in heart rate during the first 15 min of floatation may be related to changes in state anxiety, but as the model described above considers heart rate over the entire course of floatation it may miss this relationship. The interaction between serenity and breathing rate is also counterintuitive but may have a simple explanation. On the surface, it would seem that individuals with higher breathing rates over the course of floatation had the largest increase in serenity, which would be contrary to the view that lower breathing rates are associated with relaxation (Chen et al., 2017). However, a closer examination reveals that anxious participants had higher breathing rates overall as compared to non-anxious comparison participants; thus, higher breathing rates may act as a stand-in for anxious participants in this model. In other words, the individuals who showed the greatest increase in serenity following floatation were anxious participants, who naturally had higher breathing rates.

Drawing these results together, frequency domain HRV data from this experiment suggest that Floatation-REST results in a shift to higher vagal modulation *via* decreased sympathetic arousal, particularly in the sympathetic output from the baroreflex. The concomitant reductions in blood pressure and breathing rate may be related to the above changes, and this pattern of decreased sympathetic arousal is in line with prior studies showing a significant post-float decrease in circulating levels of norepinephrine (Kjellgren et al., 2001; Caldwell et al., 2022). However, the lack of significant heart rate changes paired with the moderate decrease in SDNN and small decrease in RMSSD highlight that any increase in vagal activity was limited by a compensatory process that reduced the overall variability between heartbeats and allowed the heart rate to be maintained at a normal speed.

There are several important differences between the float and film condition that must be addressed to fully contextualize these findings. Body position between the two conditions was not equivalent, with participants lying supine in the float condition, but seated upright while viewing the film. Prior studies directly comparing blood pressure while supine versus sitting have found contradictory results, with some studies showing lower blood pressure while supine (Netea et al., 1998; Cicolini et al., 2011) and other studies actually showing higher blood pressure while supine (Netea et al., 2003; Sala et al., 2005; Eşer et al., 2007). Likewise, at least one study found that frequency-based metrics of HRV, including both LF and HF, were elevated when participants were resting supine, as compared to seated or standing positions, which is the opposite of our findings (Young and Leicht, 2011). In addition to changes induced by postural differences, ambient air temperature should be taken into account, as the air temperature in the float condition was much warmer than the film condition. A previous study showed a significant reduction in systolic (but not diastolic) blood pressure when sitting in a room heated to 95°F (Sollers et al., 2002); however, this same study also found increased heart rate, increased LF, and decreased HF which is the opposite of our findings. Another potential explanation for the reduction in blood pressure during Floatation-REST could be related to vasodilation caused by the warm 95°F water. Upright immersion into 97°F water has been found to reduce blood pressure and increase HF-HRV (Miwa et al., 1997; Becker et al., 2009; Hildenbrand et al., 2010), with another study showing a similar pattern during supine floating (Nishimura and Onodera, 2001). Thus, some of the cardiovascular changes found in the present study could be due to simply being immersed in warm water. However, the salinity of the water used in this experiment might not be related to the observed decrease in blood pressure, as at least one previous study found that immersion in highly saline water (from the Dead Sea) led to an increase in blood pressure as compared to freshwater immersion (Ish-Shalom and Better, 1984). Future float studies exploring the cardiovascular changes induced by Floatation-REST should consider more active comparator conditions that entail laying supine, including perhaps in a shallow pool of 95°F water but without the Epsom salt. Such comparisons could help to clarify whether the effects reported here are related to the supine body position, immersion in warm water, or immersion in supersaturated saltwater.

There are several additional limitations to the current study. While rigorous quality control checks of the physiological data were undertaken, the wireless devices that we used are relatively new and have only been validated in a few prior studies (as outlined above under physiological data collection). The breathing rate data provided by the BioPatch used a proprietary algorithm based on impedance that may not be completely comparable to the stretch-based sensors that are traditionally used for measuring respiratory rate. The additional procedures undertaken to waterproof the physiological equipment may have possibly introduced added variability into the data, although the same procedures were also used during the film condition to ensure comparability of results. Another important limitation is that the study’s sample size was underpowered to identify small HRV effect sizes, and this should be considered when interpreting the results (Laborde et al., 2017; Shaffer and Ginsberg, 2017). While it is conceivable that there would be differences in the physiological response between anxious and non-anxious participants (e.g., Chalmers et al., 2014), the study was likely underpowered to identify such differences. Nevertheless, the findings suggest that floating may induce a similar pattern of physiological change irrespective of anxiety level.

The ability to collect physiological measures during Floatation-REST opens a path to a variety of exciting future directions. First and foremost, a replication of these findings is warranted using a more active comparison condition in order to better understand whether the pattern of cardiovascular changes found in this study represent the default state of the ANS when external stimulation of the CNS is minimized. Moreover, physiological measurements should be acquired before each condition in order to ensure that any differences between conditions are not being driven by baseline differences, which is another limitation of the current study. It will also be important to gain a better understanding of how long the cardiovascular effects last after a float session is over in order to inform the design of future longitudinal float studies. For example, the significant decreases in blood pressure raise the possibility of testing participants with hypertension to examine the longevity of floating’s hypotensive effects (Fine and Turner, 1982). Future experiments could also pair Floatation-REST with reactionary stress experiments to provide insight into how floatation might buffer the body’s response to stress, including stress-related effects on HRV (Levine et al., 2016). The results and methodology presented here provide an important foundation for these future investigations into Floatation-REST.

## Data availability statement

The raw data supporting the conclusions of this article will be made available by the authors, without undue reservation.

## Ethics statement

The studies involving human participants were reviewed and approved by the Western Institutional Review Board. The patients/participants provided their written informed consent to participate in this study. Written informed consent was obtained from the individual(s) for the publication of any potentially identifiable images or data included in this article.

## Author contributions

MF, OA, WS, JS, CW, SK, and JF contributed to the conception and design of the study. TP, OA, JN, and JF cleaned and organized the dataset. MF and H-WY performed the statistical analysis. MF, TP, and JF wrote the first draft of the manuscript. TF and JL helped write sections of the manuscript. All authors contributed to manuscript revision, read, and approved the submitted version.

## Footnotes

1
www.projectredcap.org
