# Supplementary material for: Exploring the acute cardiovascular effects of Floatation-REST
Source: Front Neurosci. 2022 Dec 9;16:995594. doi: 10.3389/fnins.2022.995594 (PMC9780456; doi:10.3389/fnins.2022.995594)
Supplement: Supplementary file 1 [file Data_Sheet_1.docx]

**SUPPLEMENTARY FIGURES**

**
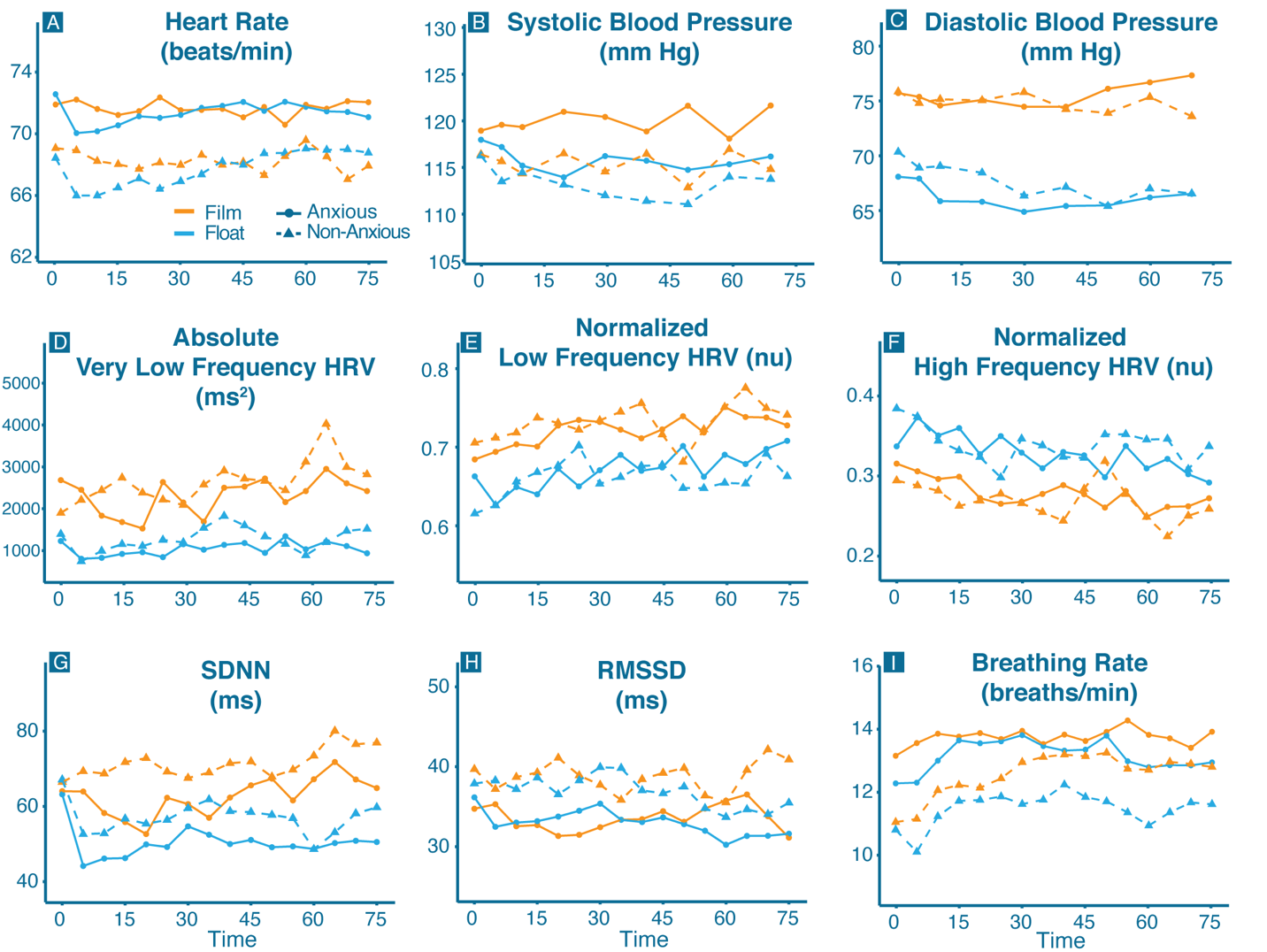
**

**Figure S1. Cardiovascular effects of Floatation-REST as compared to the film condition broken down by group.** Mean physiological response is shown for each group (solid line=anxious group; dashed line=non-anxious group) and condition (blue=float; orange=film) for (A) heart rate, (B) systolic blood pressure, (C) diastolic blood pressure, (D) absolute very low frequency HRV, (E) normalized low frequency HRV, (F) normalized high frequency HRV, (G) SDNN, (H) RMSSD, and (I) breathing rate. The x-axis represents time (in minutes) since the start of the float or film. With the exception of blood pressure (which were single point measurements), data are graphed in 5-minute bins such that timepoint 0 is the average from 0-5 minutes, and timepoint 75 is the average from 75-80 minutes. There were no significant between-group differences for any of the measures with the exception of breathing rate.

**
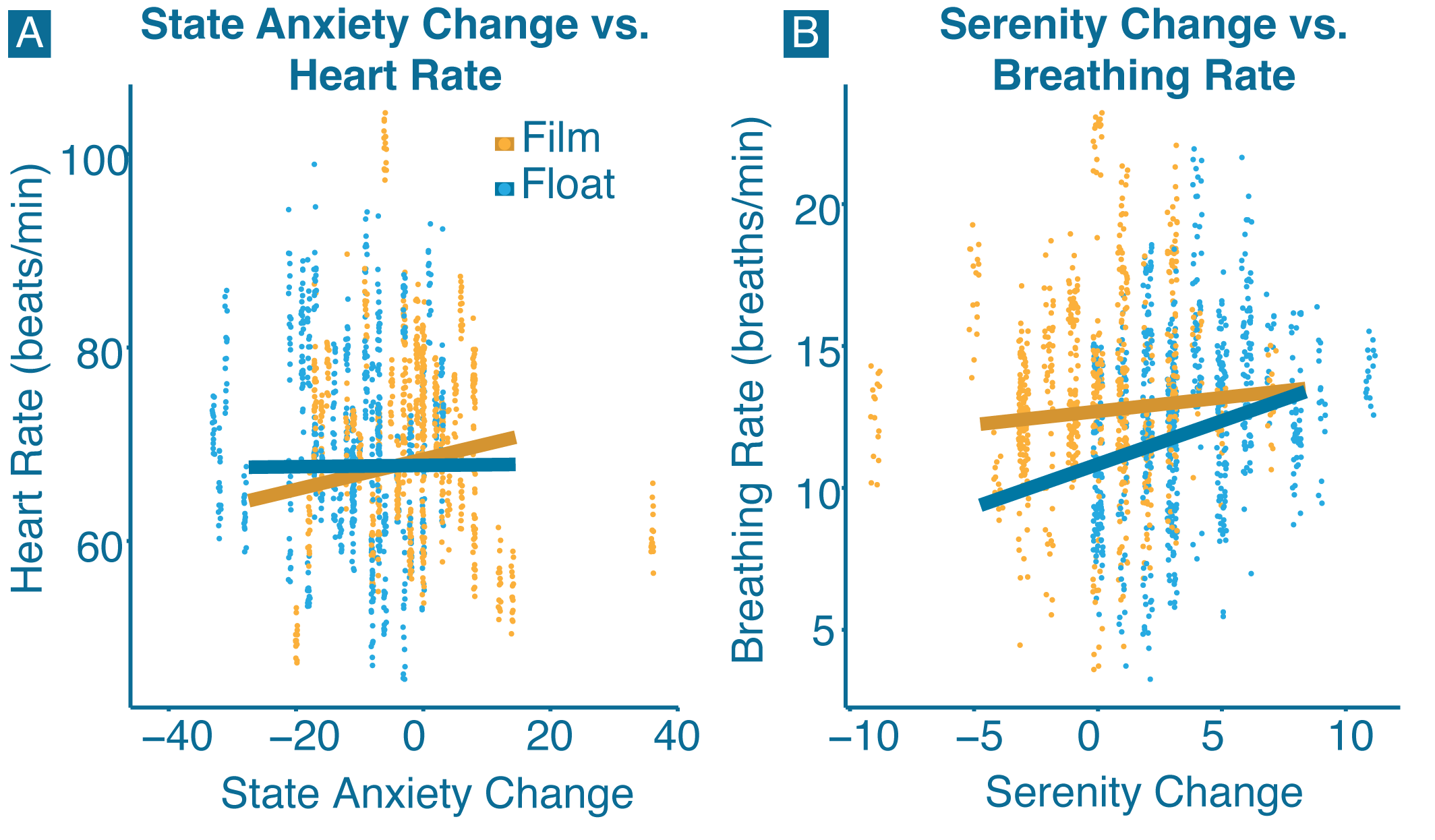
**

**Figure S2. Significant interactions between heart rate, breathing rate, and change in state anxiety and serenity.** Each point represents the average heart rate or breathing rate over a 5-minute bin for an individual participant, and each trend line shows the correlation between heart rate or breathing rate and change in state anxiety and serenity broken down for each condition (orange = film; blue = float). Significant interactions (p < .001) were found between (A) state anxiety change and heart rate, and (B) serenity change and breathing rate.

**Figure S3 - Anxious group flow diagram**

Eligible for participation (n= 43)

Randomized to float (n = 20) or film (n = 19) intervention

Received both interventions (n = 37)

Did not receive both interventions (n = 2)

Participant had adverse psychological event, unrelated

to study, prior to float (n = 1)

Participant discontinued study prior to film (n = 1)

**Allocation**

**HRV Analysis**

**Enrollment**

**Blood Pressure Analysis**

**Heart Rate Analysis**

**Breathing Rate Analysis**

• Excluded due to injury (n = 1)

• Did not show up for appointment (n = 1)

• Declined to participate (n = 2)

Analyzed (n = 36)

Lost Bluetooth connection (n = 1)

Analyzed (n = 30)

Low heart rate confidence (n = 5)

Error in analysis script (n = 2)

Analyzed (n = 29)

Low heart rate confidence (n = 5)

Error in analysis script (n = 2)

Corrupted HRV data (n = 1)

**Figure S4 - Non-anxious** **comparison group flow diagram**

Analyzed (n = 20)

Analyzed (n = 17)

Low heart rate confidence (n = 1)

Corrupted ECG data (n = 1)

Failed manual inspection (n = 1)

**Blood Pressure Analysis**

**Breathing Rate Analysis**

**HRV Analysis**

**Heart Rate Analysis**

Allocated to float (n = 13) or film (n = 7) intervention

Received both interventions (n = 20)

Did not receive both interventions (n = 0)

Eligible for participation (n = 22)

**Allocation**

**Enrollment**

• Excluded due to injury (n = 0)

• Did not show up for appointment (n = 0)

• Declined to participate (n = 2)

**SUPPLEMENTARY TABLES**

**Table S1. Criteria for entry into the initial float study for anxious participants.**

| **Inclusion Criteria** | **Exclusion Criteria** |
| --- | --- |
| 1. DSM-IV diagnosis of an anxiety disorder (generalized anxiety disorder, social anxiety disorder, panic disorder, agoraphobia) and/or posttraumatic stress disorder 2. Overall Anxiety Severity and Impairment Scale (OASIS) score ≥ 8 3. Anxiety Sensitivity Index (ASI-3) total score ≥ 30 4. If taking medication, must be stably medicated prior to participation (defined as having taken the medication for 6 weeks or longer) 5. Between 18-55 years of age 6. No prior Floatation-REST experience | 1. Comorbid bipolar disorder or schizophrenia 2. Active suicidality with intent or plan 3. Currently receiving inpatient treatment 4. Current substance use disorder ≥ moderate 5. History of neurological conditions 6. Any skin conditions or open wounds that could cause pain when exposed to saltwater 7. Inability to swim or lay comfortably in a shallow pool of water |

**Table S2. Criteria for entry into the initial float study for non-anxious participants.**

| **Inclusion Criteria** | **Exclusion Criteria** |
| --- | --- |
| 1. OASIS score < 4 2. ASI-3 score < 13 3. Between 18-55 years of age 4. No prior Floatation-REST experience | 1. Any clinical diagnosis on the DSM-IV as determined by the Mini International Neuropsychiatric Interview (MINI Version 6.0) 2. History of any neurological or psychiatric issues 3. Taking any psychotropic medications or drugs 4. Any skin conditions or open wounds that could cause pain when exposed to saltwater 5. Inability to swim or lay comfortably in a shallow pool of water |
